# Free-breathing myocardial T2* mapping using GRE-EPI and automatic Non-rigid motion correction

**DOI:** 10.1186/s12968-015-0216-z

**Published:** 2015-12-23

**Authors:** Ning Jin, Juliana Serafim da Silveira, Marie-Pierre Jolly, David N. Firmin, George Mathew, Nathan Lamba, Sharath Subramanian, Dudley J. Pennell, Subha V. Raman, Orlando P. Simonetti

**Affiliations:** Siemens Medical Solutions USA, Inc, 460 West 12th Ave, Room 311, OH 43210 Columbus, OH USA; Davis Heart & Lung Research Institute, The Ohio State University, Columbus, OH USA; Imaging & Computer Vision, Siemens Corporation, Corporate Technology, Princeton, NJ USA; NIHR Cardiovascular Biomedical Research Unit, Royal Brompton and Harefield NHS Trust, London, UK; National Heart and Lung Institute, Imperial College, London, UK; Department of Internal Medicine – Division of Cardiovascular Medicine, The Ohio State University, Columbus, OH USA; Department of Biomedical Engineering, The Ohio State University, Columbus, OH USA; Department of Radiology, The Ohio State University, Columbus, OH USA

**Keywords:** Cardiac, T2*, Mapping, Free-breathing, Iron overload, GRE-EPI, Motion correction

## Abstract

**Background:**

Measurement of myocardial T2* is becoming widely used in the assessment of patients at risk for cardiac iron overload. The conventional breath-hold, ECG-triggered, segmented, multi-echo gradient echo (MGRE) sequence used for myocardial T2* quantification is very sensitive to respiratory motion and may not be feasible in patients who are unable to breath-hold. We propose a free-breathing myocardial T2* mapping approach that combines a single-shot gradient-echo echo-planar imaging (GRE-EPI) sequence for T2*-weighted image acquisition with automatic non-rigid motion correction (MOCO) of respiratory motion between single-shot images.

**Methods:**

ECG-triggered T2*-weighted images at different echo times were acquired by a black-blood, single-shot GRE-EPI sequence during free-breathing. A single image at a single TE is acquired in each heartbeat. Automatic non-rigid MOCO was applied to correct for in-plane respiratory motion before pixel-wise T2* mapping. In a total of 117 patients referred for clinical cardiac magnetic resonance exams, the free-breathing MOCO GRE-EPI sequence was compared to the breath-hold segmented MGRE approach. Image quality was scored independently by 2 experienced observers blinded to the particular image acquisition strategy. T2* measurements in the interventricular septum and in the liver were compared for the two methods in all cases with adequate image quality.

**Results:**

T2* maps were acquired in all 117 patients using the breath-hold MGRE and the free-breathing MOCO GRE-EPI approaches, including 8 patients with myocardial iron overload and 25 patients with hepatic iron overload. The mean image quality of the free-breathing MOCO GRE-EPI images was scored significantly higher than that of the breath-hold MGRE images by both reviewers. Out of the 117 studies, 21 breath-hold MGRE studies (17.9 % of all the patients) were scored to be less than adequate or very poor by both reviewers, while only 2 free-breathing MOCO GRE-EPI studies were scored to be less than adequate image quality. In a comparative evaluation of the images with at least adequate quality, the intra-class correlation coefficients for myocardial and liver T2* were 0.868 and 0.986 respectively (p < 0.001), indicating that the T2* measured by breath-hold MGRE and free-breathing MOCO GRE-EPI were in close agreement. The coefficient of variation between the breath-hold and free-breathing approaches for myocardial and liver T2* were 9.88 % and 9.38 % respectively. Bland-Altman plots demonstrated good absolute agreement of T2* in the interventricular septum and the liver from the free-breathing and breath-hold approaches (mean differences -0.03 and 0.16 ms, respectively).

**Conclusion:**

The free-breathing approach described for T2* mapping using MOCO GRE-EPI enables accurate myocardial and liver T2* measurements and is insensitive to respiratory motion.

## Background

Cardiac failure and arrhythmia induced by myocardial iron overload is the leading cause of death in thalassemia major patients and in other patients who receive chronic blood transfusions [1, 2]. Since iron induced cardiomyopathy is treatable and reversible if intensive chelation treatment is appropriately utilized [3–5], a reliable technique to quantify the extent of myocardial iron deposition is critical to assess the risk of cardiac complications and to effectively manage these patients. In the presence of iron, T2* is shortened and inversely related to tissue iron levels; as a result, myocardial T2* measurement is becoming widely used in the assessment of patients at risk for cardiac iron overload [6–8]. T2* values can be derived from a series of T2*-weighted gradient echo images sampled at different echo times (TE). Myocardial T2* below 20 ms has been shown to indicate iron overload in the heart, and overload is considered to be severe when T2* is under 10 ms as shown by the associated increased risk of developing congestive cardiac failure within the ensuing 12 months [6, 9].

Conventionally, T2* in the heart is measured in a region of interest (ROI) manually delineated in the interventricular septum to avoid the susceptibility gradients that can be severe in other segments of the left ventricular myocardium. The mean pixel value within the septal ROI of each T2*-weighted image is calculated, and then a mono-exponential decay curve is fit to these mean values across the multiple TE images. Pixel-wise T2* mapping is another approach that is gaining popularity based on the recent success of similar approaches to myocardial T1 mapping and T2 mapping. Pixel-wise mapping involves curve fitting on individual pixels; while this process is more prone to noise than ROI fitting, it can be totally automated and recent studies comparing region-based and pixel-wise methods for cardiac T2* quantification have demonstrated that pixel-wise T2* mapping reduces inter- and intra-observer variability [10].

A breath-hold, ECG-triggered, segmented, multi-echo gradient echo (MGRE) sequence is commonly used to acquire the series of images at multiple echo times needed to quantify myocardial T2* [6–8, 11]. As with any segmented k-space acquisition, data are acquired over multiple heart beats and patient breath-hold is required to avoid respiratory motion artifacts. Unfortunately, this strategy fails in patients unable to breath-hold. As is the case for other cardiovascular magnetic resonance (CMR) applications like cine and late gadolinium enhancement imaging, an alternative free-breathing approach would be useful for myocardial T2* measurement in patients unable to breath-hold. Moreover, myocardial iron overload could occur at a very early age in children who have hereditary thalassemia and receive chronic blood transfusions [12, 13]. For those children, their first CMR evaluation could be as early as 6 years old and is normally performed under general anesthesia. The free-breathing approach offers the potential to scan children with minimal or no anesthesia.

Free-breathing methods for myocardial relaxation parameter mapping have been previously described using the combination of single-shot image acquisition and non-rigid motion correction (MOCO). Single-shot image acquisition, which acquires an entire image within one heartbeat, is inherently less sensitive to artifacts from respiratory and cardiac motion, while non-rigid MOCO ensures proper alignment of images from different respiratory positions to enable signal averaging and pixel-wise curve fitting. These methods have been successfully applied in myocardial T1 [14, 15] and T2 mapping [16, 17]. Recently, Kellman et al. [18] described a technique for free-breathing myocardial T2* mapping using a similar approach based on MOCO single-shot highly accelerated MGRE acquisition with multiple averages to improve signal-to-noise ratio (SNR). However, because this technique acquires the data for multiple images in an interleaved fashion within each heartbeat, the temporal resolution is poor (345 ms) and therefore may be prone to cardiac motion artifact in patients with higher heart rate.

In this work, we propose a different approach for free-breathing myocardial T2* mapping using a single-shot gradient-echo echo-planar imaging (GRE-EPI) sequence for T2*-weighted image acquisition, and automatic non-rigid MOCO tailored for myocardial T2* mapping. A single image at a single TE is acquired in each heartbeat, using a segmented EPI readout to greatly accelerate each T2*-weighted image acquisition, therefore reducing the potential sensitivity to cardiac and respiratory motion artifact. Furthermore, because each T2*-weighted image at each different echo time is acquired independently in a single heart beat; the choice of echo times used in the curve fitting is more flexible than in the multi-echo GRE approach; i.e., the TEs are not restricted by echo spacing. We compared myocardial and hepatic T2* measurements using free-breathing GRE-EPI and breath-hold MGRE approaches in a cohort of patients referred for clinical CMR in two different imaging centers, The Ohio State University Richard M. Ross Heart Hospital (OSU) in Columbus, OH and The Royal Brompton Hospital (RBH) in London, England. We hypothesize that the combination of single-shot GRE-EPI and motion correction will enable free-breathing myocardial T2* mapping with results comparable to the standard, breath-hold, MGRE, segmented k-space technique.

## Methods

### Imaging sequences

**Fig. 1:**
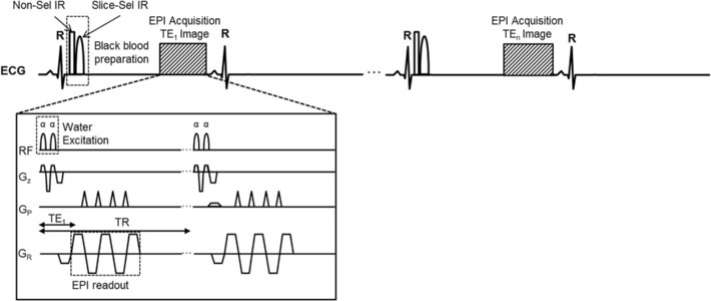
Sequence diagram for a black-blood, single-shot GRE-EPI which acquires a series of ECG-triggered T2*-weighted images at different echo times (TEs) during free-breathing

A black-blood, single-shot GRE-EPI sequence (Fig. 1.) was developed to acquire a series of ECG-triggered T2*-weighted images at different TEs during free-breathing. The sequence was implemented with an echo-train-length (ETL) of 5 and a centric k-space reordering scheme [19] to minimize the shortest achievable TE. Seven TEs ranging from 1.9 ms up to a maximum of 14 ms were acquired; a repetition time (TR) of 19.2 ms was used for all images; this was the minimum TR required to accommodate the longest TE. A 10°, rapid 1-1 binomial water excitation pulse [20] was used to suppress off-resonance artifacts caused by fat. A dark-blood, double inversion recovery preparation was applied at the R-wave trigger and the inversion time set to extend into diastole to minimize the contamination of myocardial signal by the adjacent blood pool [11, 21, 22]. A GRAPPA acceleration [23] rate of 2 with 14 integrated reference lines was used to further accelerate the acquisition. An imaging matrix of 86 x 192 resulted in an acquisition window of less than 200 ms. The sequence was triggered every second cardiac cycle as is typical for sequences using the double inversion preparation to allow for magnetization recovery and more effective blood nulling. Each of the seven T2*-weighted images was acquired in mid to late diastole in a single heartbeat; this was repeated 4 times and averaging was used following motion correction to increase SNR. The total acquisition time was 64 heartbeats with all of the data acquired during free breathing.

A breath-hold, ECG-triggered, black-blood, segmented MGRE sequence with gradient fly-back for mono-polar readout was used as the reference standard. In every TR, eight echoes were acquired to form eight T2*-weighted images, each at a different echo time. Seven to nine k-space lines (segments) were acquired in each cardiac cycle, resulting in a typical scan time of ten to thirteen heartbeats. Two different imaging matrix sizes were used depending on the site where the data were acquired. The detailed imaging protocols are listed in Table 1.

**Table 1:** Imaging protocols

	Breath-hold Segmented MGRE		Free-breathing GRE-EPI
	OSU	RBH	OSU and RBH
Acquisition Matrix	160 × 90	256 × 84	192 × 86
Slice thickness (mm)	10	10	10
Flip angle (degree)	18	20	10
TEs (ms)	1.9, 3.5, 5.1, 6.7, 8.3, 9.9, 11.5, 13.1	1.6, 3.8, 6, 8.3, 10.5, 12.7, 14.9, 17.2	1.9, 3, 5, 7, 9, 11, 14
TR (ms)	15	20	19.2
Bandwidth (Hz/Pixel)	814	814	1860
ETL	1	1	5
Segments	7	9	10
Acquisition window	105	180	192
Parallel acceleration	None		GRAPPA rate 2 with integrated reference lines
ECG triggering (heart beat)	1	1	2
Measurements	1	1	4
Total acquisition (heart beats)	13	10	64

### Image processing

**Fig. 2:**
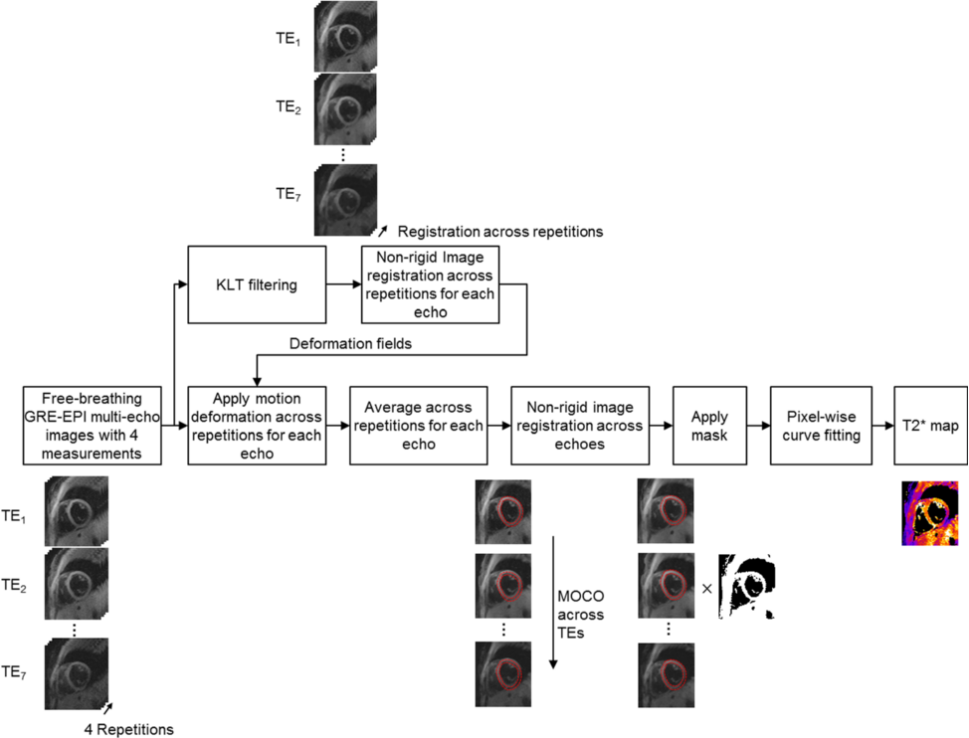
The workflow to generate the T2* map from free-breathing, T2*-weighted images acquired using the ECG-triggered, dark-blood, single-shot GRE-EPI sequence

Free-breathing, T2*-weighted images from the ECG-triggered, dark-blood, single-shot GRE-EPI sequence were motion corrected using automatic non-rigid image registration [24] to correct for in-plane respiratory motion. MOCO was applied in two steps: first across the four images acquired at each TE prior to averaging, and then across the averaged images at different TEs prior to generating the T2* map. The workflow is summarized in Fig. 2. All of the acquired T2*-weighted images (across all TEs and repetitions) were first passed through a Karhunen-Loève Transform (KLT) filter [25, 26] to enhance SNR prior to image registration. The KLT-filtered images were then registered using a non-rigid image motion correction algorithm [24], with the first measurement of the 4 repetitions at each TE selected as the reference image. The algorithm estimates the deformation field that minimizes the sum of squared differences (SSD) between the reference and target images. The deformation was modeled as a smooth vector field that gives for each pixel in the reference image its corresponding location on the target images. The algorithm recovers the deformation by applying small displacements, incrementally maximizing the similarity criterion. To speed-up the convergence and avoid local optima, an iterative multi-scale coarse-to-fine image pyramid was created, which contained a set of images with low-to-high image resolutions in different layers. Once the deformation field was estimated, it was used to warp the corresponding unfiltered images using a subpixel spline-based interpolation. Applying the KLT filter before image registration improved image SNR and hence the robustness of the estimation of the deformation fields, while warping the raw unfiltered images prevented any unwanted image blurring introduced by the filtering process from propagating through to the T2* map. After image registration, the four motion-corrected images acquired at each TE were averaged. The SSDs between the motion-corrected images and the target image were calculated and served as weighting factors for averaging; the inverse of the SSD was used as the weighting factor in order to minimize the impact of images corrupted by arrhythmias or poor ECG gating. The intermediate result at this stage was a motion-corrected, averaged image at each TE. The MOCO algorithm was then applied again to the averaged images across the different TEs, with the image at the shortest TE (i.e., the image with highest signal) selected as the reference image. A deformation field was estimated to maximize the local cross-correlation (LCC) between the reference and uncorrected images. The LCC criterion was used instead of SSD as it is more robust in the presence of image intensity and contrast changes, as is the case for images with varying TEs. The end result of this two-stage process was a series of motion corrected, averaged images aligned across the different TEs in preparation for T2* map calculation.

To remove noise regions (air in the lungs and outside of the body) in the T2* maps, an automatic binary mask was generated based on the maximum intensity projection across the images at different TEs using the iterative Otsu threshold [27]; this technique performs clustering-based thresholding to separate an image into foreground (mask value = 1) and background (mask value = 0, i.e., noise) components. The images were multiplied by this mask prior to T2* fitting to reduce calculation time. A T2* map was then generated pixel-wise by fitting a two-parameter mono-exponential model S(TE_i_) = S(0) exp(-TE_i_/T2*), where S(TE_i_) is the signal intensity at the i^th^ echo and TE_i_ is the echo time. A robust iteratively re-weighted fitting was used [28, 29], in which the signal at each TE is iteratively weighted to reflect its fidelity to a mono-exponential decay curve. This method reduces the influence of points further from the ideal relaxation curve. The entire image processing (motion correction, filtering, averaging, and pixel-wise fitting) was done automatically on the scanner.

### Patient experiments

Imaging was performed on two identical 1.5 T clinical scanners (MAGNETOM Avanto, Siemens Healthcare, Erlangen, Germany), with maximum gradient amplitude of 45 mT/m, and maximum slew rate of 200 mT/m/ms. Body matrix array and spinal array coils were used for signal reception.

A total of 117 patients (56 males) 47.7 ± 17.1 years, ranging from 9 to 82, referred for clinical CMR exams that included evaluation of myocardial T2* were studied to evaluate the effectiveness of this new technique and to compare image quality and quantitative T2* results with the standard, segmented k-space method. Eighty-eight of the patients were referred for evaluation of cardiomyopathy and were imaged at OSU. Twenty-nine patients with known thalassemia referred to CMR specifically for evaluation of myocardial and hepatic iron were imaged at RBH. All patients underwent T2* imaging using both the conventional segmented MGRE sequence during breath-hold, and the new MOCO GRE-EPI sequence during free-breathing. Identical scan parameters were used for the MOCO GRE-EPI sequence at both centers. The breath-hold MGRE protocol used at RBH had a somewhat higher image matrix than the protocol used at OSU (256 × 84 vs. 160 x 90); this minor difference only reflected the standard clinical scan parameters employed by each site. A single mid-ventricular short axis slice was acquired in all patients. The study was approved by the local Institutional Review Boards in both sites; informed consent was waived.

### Image quality comparison

Qualitative comparisons were made between the images acquired using the breath-hold MGRE and the free-breathing GRE-EPI sequences. For each acquisition strategy in each patient, the first 7 T2*-weighted images (with echo times ranging from 1.9 ms to 14 ms) were displayed on a single slide for the purpose of image quality scoring. The order of display was randomized with regard to both subject number and acquisition strategy. Image quality was scored independently by 2 experienced observers blinded to the particular image acquisition strategy. A 5-point scoring system was used to assess image quality based on artifact level: 1 - Very poor image quality with unusable images; 2 - Less than adequate image quality with substantial artifact; 3 - Adequate image quality with moderate artifact; 4 - Good image quality with minimal artifacts; 5 - Excellent image quality with no significant artifact. The image quality scores for the breath-hold and free-breathing techniques from the two reviewers were compared separately using the pairwise Wilcoxon signed rank test with a significance level of 0.05.

### Quantitative T2* results comparison

In only those cases in which both techniques were judged to have at least adequate image quality (image quality score ≥ 3) by both reviewers, the mean T2* was measured in the interventricular septum and in the liver in each patient by manually drawing ROIs directly on the T2* maps. A comparison between the mean T2* values derived from the breath-hold and free-breathing approaches for both liver and myocardium in each patient was performed by calculating the intra-class correlation coefficient (ICC) [30] with two-way mixed effect model; ICC values near 1 indicate that the two methods yielded consistent results, while values near 0.5 indicate unreliable measurements. A coefficient of variation (CoV), defined as the standard deviation of the difference between the T2* from the two methods divided by the average T2* from the two methods across all the patients, was also calculated to measure the dispersion between the breath-hold and free-breathing approaches. The T2* results were further analyzed using the Bland-Altman method to visualize the agreement between the two methods. For each patient, the breath-hold T2* was compared with the corresponding free-breathing T2* using a pairwise Student’s *t*-test with significance level of 0.05.

Calibration curves relating liver T2* to liver iron content have been reported [31–33] and allow for estimation of liver iron concentration (LIC). Based on the equation provided in [33], LIC was estimated as LIC = 28.02/T2* - 0.454.

## Results

**Fig. 3:**
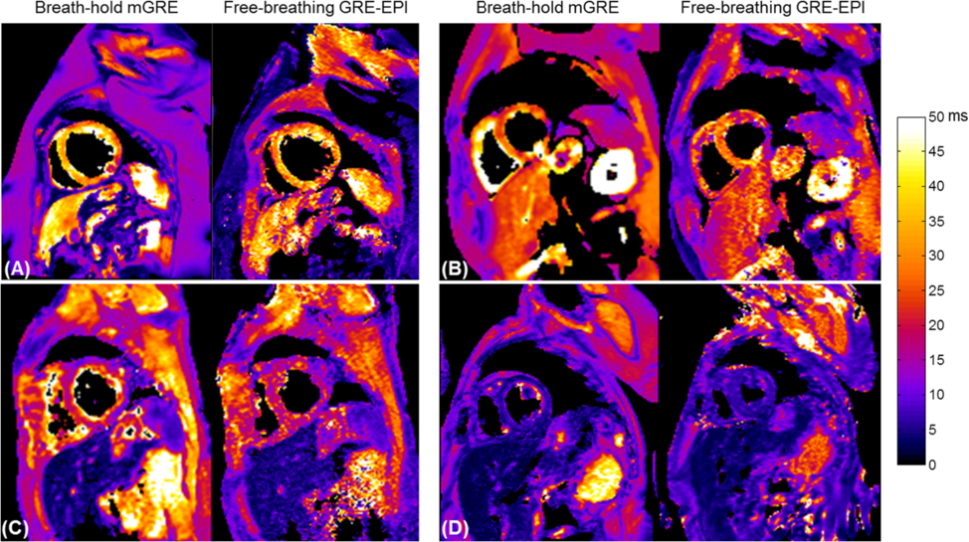
Representative examples of T2* maps in four patients (**a**–**d**) acquired using breath-hold MGRE and free-breathing MOCO GRE-EPI. All four of these patients were able to hold their breath successfully during the breath-hold MGRE exam and both techniques produced T2* maps of good image quality

T2* maps were acquired in all 117 patients using the breath-hold MGRE and the free-breathing MOCO GRE-EPI approaches. Eight patients presented myocardial iron overload, as determined by the standard MGRE technique. Myocardial overload was mild in 6 cases (14 ms ≤ T2* < 20 ms), moderate in 1 case (10 ms ≤ T2* < 14 ms,) and severe in 1 case (T2* < 10 ms). Hepatic iron overload was present in 25 patients. Mild hepatic iron burden (3.8 ms ≤ T2* < 11.4 ms, 2.0 mg/g ≤ LIC < 7.0 mg/g) [34] was present in 12 patients, moderate overload (1.8 ms ≤ T2* < 3.8 ms, 7.0 mg/g ≤ LIC < 15.0 mg/g) [34] was present in 9 patients and severe overload was present in 4 patients (T2* < 1.8 ms, LIC ≥ 15.0 mg/g) [34]. All patients who had myocardial iron overload also had hepatic iron overload. Representative examples of T2* maps acquired using the conventional breath-hold MGRE and the new free-breathing MOCO GRE-EPI in 4 patients are shown in Fig. 3. All four of these patients were able to hold their breath successfully during the breath-hold MGRE exam. In these examples, both techniques produced T2* maps of good image quality and myocardial and hepatic T2* were comparable between the techniques. Patient 1 and patient 2 (Fig. 3a and b) had normal hepatic T2* (T2* > 11.4 ms) and myocardial T2* (T2* > 20 ms). Patient 3 (Fig. 3c) had both mild hepatic (3.8 ms ≤ T2* < 11.4 ms) and myocardial iron (14 ms < T2* < 20 ms). Patient 4 (Fig. 3d) had moderate hepatic (1.8 ms ≤ T2* < 3.8 ms) and severe myocardial iron overload (T2* < 10 ms).

**Fig. 4:**
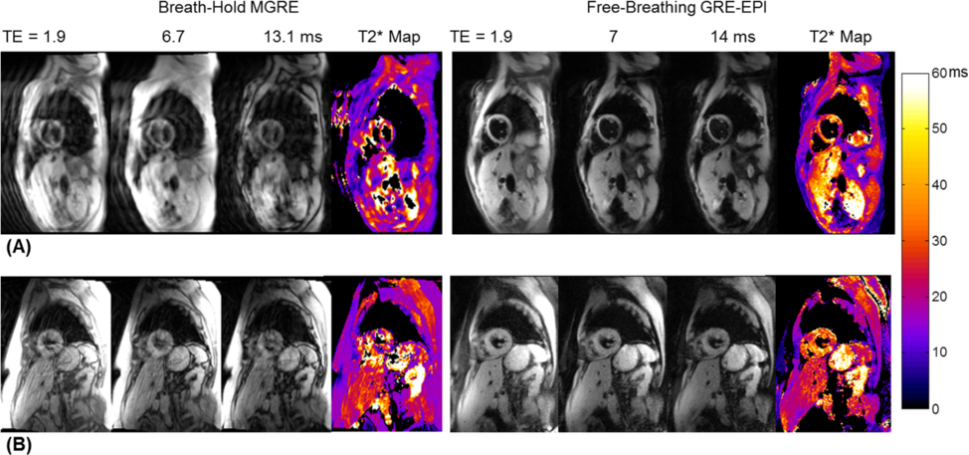
Examples of the T2*-weighted source images and their corresponding T2* maps in two patients (top and bottom) who failed to hold their breath during the breath-hold MGRE T2* scan: Severe ghosting and image blurring artifacts caused by respiratory motion during image acquisition are evident in the T2*-weighted images acquired with segmented MGRE; the corresponding T2* maps were also corrupted by respiratory motion (**a** and **c**), while the single-shot MOCO GRE-EPI acquisition effectively froze respiratory motion during the image acquisition and produced artifact-free T2* maps during free-breathing (**b** and **d**)

Figure 4 shows examples in two patients who failed to hold their breath during the breath-hold MGRE T2* scan. T2*-weighted source images acquired at three TEs and their corresponding T2* maps are shown for both the breath-hold (Fig. 4a and c) and the free-breathing (Fig. 3b and d) techniques. Both patients had normal hepatic and myocardial T2* with no indication of iron overload. Severe ghosting and image blurring artifacts caused by respiratory motion during image acquisition are clearly evident in the T2*-weighted images acquired with segmented MGRE; the corresponding T2* maps were also corrupted by respiratory motion. In Fig. 4a, even though the interventricular septum is still visible in the T2* map from the breath-hold segmented MGRE acquisition, severe T2* variations are observed. On the other hand, in this patient the single-shot MOCO GRE-EPI acquisition effectively froze respiratory motion during the image acquisition and produced artifact-free T2* maps during free-breathing.

**Fig. 5:**
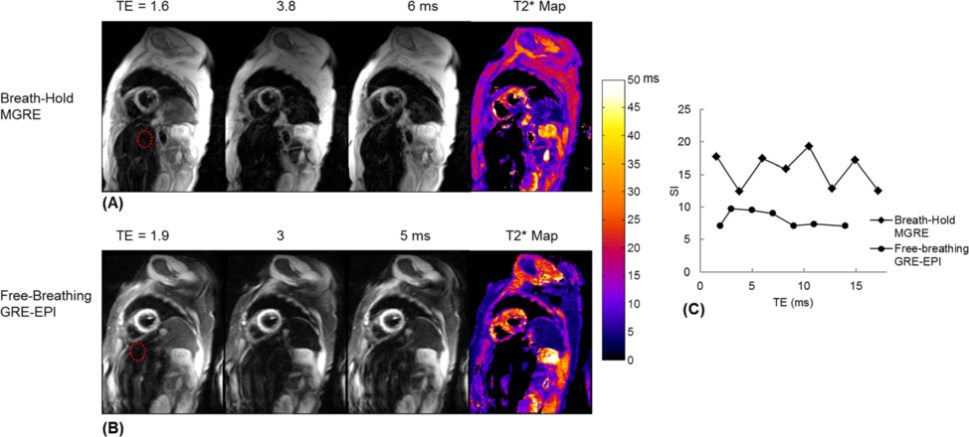
One example of T2*-weighted source images of the first three echoes and their resulting T2* maps in a patient with normal heart and severe hepatic iron overload. Images were acquired using breath-hold MGRE (**a**) and free-breathing GRE-EPI approaches (**b**) Both techniques failed to provide T2* quantification in the liver. (**c**) Curves of mean signal intensity within the ROI in the liver (red circle in **a** and **b**) vs. echo times, showing that the signal is below the noise level even in the image from the earliest echo

In the four patients with severe hepatic iron overload, both breath-hold and free-breathing techniques did not provide reliable T2* measurements in the liver. Figure 5 shows one example of T2*-weighted source images of the first three echoes and their corresponding T2* maps in a patient with normal heart but severe hepatic iron overload. Images were acquired using breath-hold MGRE and free-breathing GRE-EPI approaches. There is almost no signal evident in the liver in any of the source images due to the very short T2*. Figure 5c shows a plot of the mean signal intensity within the ROI in the liver (red circle in Fig. 5a and b) vs. echo times, showing that the signal is below the noise level even in the earliest echo images. Hence, the fitting for T2* failed and T2* in the liver was automatically set to zero in the T2* maps for both techniques.

The mean image quality of the free-breathing MOCO GRE-EPI images was scored significantly higher than that of the breath-hold MGRE images by both reviewers (*p* = 0.0101 and 0.0324 respectively). The average image quality scores from reviewer #1 were 3.34 ± 1.40 and 3.72 ± 0.71 for breath-hold MGRE and free-breathing MOCO GRE-EPI, respectively. The average quality scores from review #2 were 3.82 ± 1.31 and 4.10 ± 0.74 for breath-hold MGRE and free-breathing MOCO GRE-EPI, respectively. Both reviewers agreed that image quality was more variable in the T2*-weighted images acquired using breath-hold MGRE due to the low image quality that resulted from failed breath-hold in some patients. Out of the 117 studies, reviewer #1 scored the image quality of 21 breath-hold MGRE studies (17.9 % of all the patients) to be below average or very poor (image quality score ≤ 2); while reviewer #2 scored 36 breath-hold MGRE studies (30.7 % of all the patients) to be below average or very poor, including the same 21 patients as reviewer #1. Only 2 free-breathing MOCO GRE-EPI studies were scored to be less than adequate image quality, while the MGRE images of one patient were scored to be very poor image quality by both reviewers due to failed breath-hold.

**Fig. 6:**
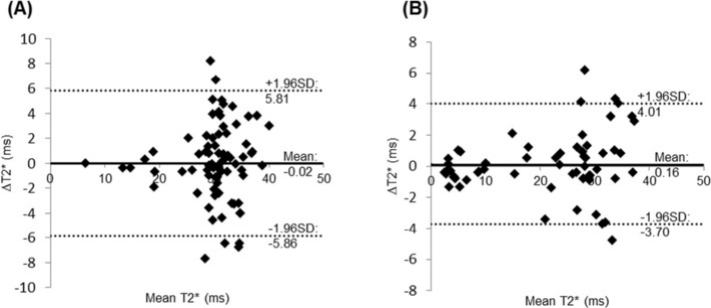
Bland-Altman plots showing the agreement for the T2* in the interventricular septum (**a**) and the liver (**b**) from free-breathing MOCO GRE-EPI and breath-hold MGRE measurements

The 37 patients whose image quality was scored as less than adequate image quality by at least one reviewer for either technique were excluded from the comparison of quantitative T2* results. Out of the 80 patients with adequate image quality from both breath-hold MGRE and free-breathing MOCO GRE-EPI techniques, 17 patients did not have a large enough homogeneous liver region within the ventricular short axis image to permit measurement. 4 patients had severe hepatic iron overload and both techniques failed to measure T2* in the liver. Thus, the T2* values measured in the interventricular septum and in the liver using both techniques were compared in 80 patients and 57 patients, respectively. The ICCs, for myocardial and hepatic T2*, were 0.868 and 0.986 respectively (*p* < 0.001), indicating that the T2* measured by breath-hold MGRE and free-breathing MOCO GRE-EPI were very consistent. The CoVs between the breath-hold and free-breathing approaches for myocardial and hepatic T2* were 9.88 % and 9.38 % respectively. As demonstrated in the Bland-Altman plots shown in Fig. 6a and b, T2* in the interventricular septum (Fig.6a) and the liver (Fig. 6b) from free-breathing MOCO GRE-EPI showed good absolute agreement with the breath-hold measurements without significant bias. Likewise, the pairwise *t*-test showed that there were no significant differences between the breath-hold and free-breathing approaches in either myocardial or hepatic T2* (*p* = 0.937 and 0.598 respectively).

## Discussion

Myocardial T2* is sensitive to iron deposition in the heart and has become an important diagnostic tool used to assess the risk of cardiac complications and to guide appropriate iron-chelation therapy in thalassemia patients. However, the segmented MGRE sequence commonly used for myocardial T2* quantification is exquisitely sensitive to respiratory motion and requires a steady breath-hold during the image acquisition. Thus, the standard technique may not be feasible in anyone unable to comply with breath-hold commands. In 117 patients who underwent T2* mapping, we found that 17.9 % of the T2* measurements using the standard segmented sequence were scored by both reviewers to be non-diagnostic due to failed breath-hold. To overcome this limitation in the applicability of the conventional segmented MGRE acquisition, we developed a new free-breathing myocardial T2* mapping approach that combines a single heartbeat T2*-weighted image acquisition with automatic correction of respiratory motion between single-shot images. The measured T2* in the myocardial septum and the liver were in good agreement with the standard breath-hold segmented MGRE technique in those patients who were capable of breath-holding, and the new free-breathing technique produced higher image quality than the standard method in patients unable to breath hold.

Different from the segmented k-space approach that acquires the data for each image segmentally across multiple heartbeats MOCO GRE-EPI acquires the entire k-space for a single T2*-weighted image in a single heartbeat; this approach is directly analogous to the methods for myocardial T1 *[14, 15] and T2 [16] mapping based on single-shot imaging with motion correction that have recently become popular. This free-breathing MOCO GRE-EPI T2* mapping approach could be particularly beneficial to young children receiving multiple transfusions and whose myocardial iron concentration must be monitored frequently at a very early age. Since this new approach is motion insensitive and does not require breath-hold, it may be possible to scan children without general anesthesia or under minimal sedation. Furthermore, the new free-breathing T2* approach does not require breath-hold training before the scan, or time for the patient to catch their breath after each acquisition. This should simplify the workflow and avoid the necessity to repeat T2* measurements when breath-holding fails using conventional techniques. Thus, even though the acquisition time for the new free-breathing technique is slightly longer compared to the breath-hold approach, it may still reduce the overall MR table time and increase patient throughput. This may be particularly useful in Middle-Eastern or Asian countries where there is higher incidence of thalassemia and the MRI table time for each patient is typically limited.

The multi-shot EPI-based k-space sampling approach with an EPI factor of 5 provides the efficiency needed to achieve a very short acquisition window (< 200 ms) without pushing the parallel acceleration beyond a factor of 2 thereby avoiding excessive g-factor SNR loss. This acquisition speed is fast enough to avoid respiratory induced artifacts, and motion correction is utilized to compensate for in-plane respiratory motion between images secondary to variable diaphragm positions during acquisition. Compared to the free-breathing T2* mapping technique recently described in [18], which uses highly undersampled single-shot MGRE with respiratory motion corrected averaging, the temporal footprint of each T2*-weighted image using our technique is much shorter (<200 ms vs. 345 ms). This shorter acquisition window should render our technique less sensitive to motion-induced image blurring or the loss of spatial resolution, especially in subjects with higher heart rates; however a head-to-head comparison of these two free-breathing techniques is required to verify whether this theoretical advantage is significant. Furthermore, our single heartbeat GRE-EPI sequence provides more flexibility in defining TEs. In the conventional MGRE sequence, as well as the other recently described free-breathing sequence [18], multiple gradient echoes are sampled after each RF excitation, with the choice of TEs restricted by a fixed echo spacing. In our proposed free-breathing GRE-EPI sequence, each T2*-weighted image is acquired independently in a single heartbeat; a centric reordered echo train permits a choice of TEs that is not limited by echo spacing. This may provide advantages in terms of optimal selection of echo times for curve fitting under conditions of very low T2* although this must be evaluated.

Automatic non-rigid image registration was used to correct for in-plane respiratory motion. Clinical myocardial T2* maps are commonly acquired in the mid-ventricular short axis view in which it has been previously demonstrated that through-plane motion is not significant [35]. Since only 4 measurements were acquired per TE and a weighted averaging based on SSD was used to improve the MOCO quality, no measurements were discarded. If long-axis imaging orientations, such as the four-chamber view or transverse view are required, through-plane respiratory motion would have to be taken into account. It has been demonstrated for T2 mapping that navigator gating and prospective slice correction combined with non-rigid registration can be used to provide complementary compensation of both through-plane and in-plane motion [16]. This same approach could be applied to the GRE-EPI sequence to avoid through-plane motion and allow for free-breathing T2* measurements in four-chamber and trans-axial orientations.

Image quality scoring was performed intentionally using the T2*-weighted source images rather than the resulting T2* maps. Motion ghosting and other artifacts tend to be more visible in the original T2*-weighted images and may not be as obvious in the T2* maps. For example, in Fig. 3a, severe ghosting artifacts are observed in the T2*-weighted images across multiple TEs, but the impact of these artifacts is less visible in the reconstructed T2* map. In this example, the interventricular septum in the T2* map appears to be relatively unaffected by artifacts, however, the source images are clearly contaminated and the resulting T2* value should not be trusted. This also emphasizes the point that when using any parameter mapping technique, the original source images should always be reviewed.

An iterative robust fitting algorithm that reweighted the signal intensity at all TEs to reflect fidelity to mono-exponential decay was used to fit the T2* curves. Pixel-wise T2* estimation using this method has been previously compared with ROI-based T2* assessment using commercially available validated software (CMRTools, Cardiovascular Imaging Solutions, Ltd., London, UK). It was found that the mean T2* within the ROI of the interventricular septum was highly correlated with the ROI-based T2* estimation [28]. Recently, alternative pixel-wise T2* curve fitting methods have been proposed to improve the accuracy and precision of T2* estimates in images with low SNR, as can be the case when the T2* is severely shortened. Feng et al. [36] applied nonlocal means filtering to reduce noise in T2*-weighted images while maintaining the intrinsic signal. This method was compared with low-pass Gaussian filtering and found to be more robust with superior noise suppression and detail preservation. Sandino et al. [37] recently extended the automatic truncation method [38] to reduce T2* overestimation due to noise bias by using an SNR-scaled image reconstruction and truncating low SNR measurements on a pixel-wise basis. Both methods are compatible with our proposed free-breathing T2* mapping approach. While beyond the scope of this study, the evaluation of different curve fitting approaches that may improve the accuracy and precision of our free-breathing technique are warranted.

The primary limitation of this study was that relatively few patients with significant myocardial iron overload were included. A larger systematic study will be necessary to compare and evaluate the performance of the conventional breath-hold and new free-breathing myocardial T2* mapping techniques in patients with low T2* in the heart. Regarding liver T2* it should be noted that both the breath-hold and free-breathing protocols failed to measure very short T2* (<1.8 ms) in patients with severe hepatic iron overload. In the breath-hold MGRE protocols used at the two test centers, the earliest echo time sampled was 1.6 or 1.9 ms and the echo spacing between TEs was 2.2 or 1.6 ms determining by the imaging matrix (256 vs. 160) and readout bandwidth (814Hz/Pixel). Due to the very fast T2* decay, as shown in Fig. 3, there are not enough points (<3) sampled at early echoes where the signal is above the noise bias. In order to quantify very short T2* in the liver with severe iron overload, it would be necessary to reduce the minimum TE and the echo spacing in the MGRE sequence by using a smaller readout matrix with increased readout bandwidth. In the free-breathing GRE-EPI sequence, centric reordering [19] of the echo train was implemented, acquiring the central portion of k-space with the first echo. A fast 1-1 binomial water excitation pulse [20] was used to suppress the off-resonance artifacts caused by fat. With this combination of centric reordering and a 1-1 binomial water excitation pulse, the minimum TE achievable by the sequence (1.9 ms) was comparable to that of the segmented MGRE protocol in this study. It might be possible to further reduce the minimum TE by using other fat suppression techniques, such as a fat saturation preparation pulse [39]. While the technique may not be accurate at extremely low liver T2*, values lower than 1.8 ms already indicate an exceptionally high level of liver iron, and accurate quantification at such high levels may not be critical. The technique is able to determine whether liver iron is extremely high, and that may already provide adequate clinical information to guide chelation therapy until the liver iron is lowered to a measureable level. However, if it is necessary to quantify liver iron at a high level where T2* fails, for example in chelation therapy clinical trials, some alternative methods such as liver R2 (rather than R2*) measurement [31, 40] or biopsy may be used. Furthermore, as our free-breathing GRE-EPI sequence acquires each T2*-weighted image at a different TE independently in a single heartbeat, the echo spacing between the echo times is no longer restricted by the readout matrix or bandwidth as in the MGRE sequence. In the future, we will explore this feature of the sequence to determine whether the measurement accuracy of very short T2* (<1.8 ms) in the severe hepatic iron overload patients is improved by reducing echo spacing and acquiring additional early echoes. The GRE-EPI acquisition can be sensitive to off-resonance artifacts, and while in this study the GRE-EPI acquisition resulted in good image quality in 115 out of 117 patients, some residual fat artifacts may be expected if B_0_ field homogeneity is poor or there is excessive adipose tissue in the FOV. Advanced, volume selective shimming procedures may be applied to further improve B_0_ field homogeneity and reduce off-resonance artifacts from fat [41] or local field inhomogeneity. It should also be noted that the GRE-EPI sequence is mixing the signal from different echo times into each image, and this might be expected to corrupt the relationship between T2* and TE, where the effective TE is defined by the echo used to sample the center of k-space. By keeping the echo train relatively short (5 echoes) and the echo spacing tight (0.9 ms), we did not observe any negative impact from incorporating multiple TEs within each image; the measured T2* was in good agreement with the standard approach when breath holding was not an issue.

## Conclusion

We have developed a technique for T2* mapping using MOCO GRE-EPI that enables accurate myocardial and hepatic T2* measurements and is insensitive to respiratory motion. The approach is fully automatic and could be especially beneficial for patients who are unable to breath-hold during the T2* exam.

## Abbreviations

CMR: Cardiovascular magnetic resonance
CoV: Coefficient of variation
ETL: Echo-train-length
GRE-EPI: Gradient-echo echo-planar imaging
ICC: Intra-class correlation coefficient
KLT: Karhunen-Loeve Transform
LCC: Local cross-correlation
LIC: Liver iron concentration
MGRE: Multi-echo gradient echo
MOCO: Motion correction
OSU: The Ohio State University
RBH: The Royal Brompton Hospital
ROI: Region of interest
SNR: Signal-to-noise ratio
SSD: Sum of squared difference
TE: Echo time
TR: Repetition time
